# Hepatitis B Core Antibody Level: A Surrogate Marker for Host Antiviral Immunity in Chronic Hepatitis B Virus Infections

**DOI:** 10.3390/v15051111

**Published:** 2023-05-03

**Authors:** Yang Shi, Zihan Wang, Shengxiang Ge, Ningshao Xia, Quan Yuan

**Affiliations:** 1 State Key Laboratory of Infectious Disease Vaccine Development, Xiang An Biomedicine Laboratory, School of Public Health, Xiamen University, Xiamen 361102, China; shiyang@stu.xmu.edu.cn (Y.S.); wzihan@stu.xmu.edu.cn (Z.W.); sxge@xmu.edu.cn (S.G.); nsxia@xmu.edu.cn (N.X.); 2 NMPA Key Laboratory for Research and Evaluation of Infectious Disease Diagnostic Technology, National Institute of Diagnostics Vaccine Development in Infectious Diseases, School of Public Health, Xiamen 361102, China

**Keywords:** hepatitis B virus, qAnti-HBc, antiviral immunity, treatment response

## Abstract

The hepatitis B virus core protein (HBcAg) is a highly immunogenic particulate antigen. Nearly all patients with persistent or resolved hepatitis B virus (HBV) infection show seropositivity for hepatitis B core antibody (anti-HBc), which appears in the early stage of infection and is mostly present for life. Traditionally, the anti-HBc is regarded as an evidential serological marker of HBV infections. In the last ten years, several studies revealed the predictive value of quantitative anti-HBc (qAnti-HBc) level in the treatment response and clinical outcome of chronic HBV infections, implying new insights into this classic marker. Overall, qAnti-HBc should be regarded as an indicator of the host’s immune response specific to HBV, which correlates with HBV-related hepatitis activity and liver pathology. This review summarized the latest understanding of the clinical values of qAnti-HBc for differentiating the CHB phase, predicting treatment response, and providing disease prognosis. Moreover, we also discussed the possible mechanism of qAnti-HBc regulation during different courses of HBV infection.

## 1. Introduction

The hepatitis B virus (HBV) is one major pathogen causing viral hepatitis in humans and putting people at high risk of death from cirrhosis and liver cancer. The virus is transmitted mainly through mother-to-child transmission, which leads to many people suffering from hepatitis B for the rest of their lives; in addition, it can be transmitted to others through blood or sexual contact [1]. Worldwide, the World Health Organization (WHO) estimates that about 296 million individuals have chronic HBV infection, and about 800,000 people die from HBV-related diseases annually [2,3,4]. The advent and wide application of the hepatitis B vaccine have greatly reduced the incidence of HBV infection in the past 40 years. However, currently used anti-HBV drugs, for either pegylated interferons (PEG-IFNs) or nucleoside/nucleotide analogues (NAs), cannot effectively eradicate the virus; huge populations with chronic HBV infection still have a high risk of liver diseases. The World Health Organization (WHO) introduced the first global health sector strategy on viral hepatitis in 2016, with the goal of eliminating viral hepatitis, including hepatitis B, as a major public health threat by 2030 [5,6]. This strategy aims to reduce new viral hepatitis infections by 90% and lower viral hepatitis-related deaths by 65%. The success of achieving this goal in regions such as Asia will depend largely on the effective control of hepatitis B. Treating chronic hepatitis B (CHB) patients remains a challenge due to the presence of covalently closed circular DNA (cccDNA) in hepatocytes. Viral cccDNA is the molecular basis for HBV persistence, which forms a stable minichromosome and serves as a viral transcriptional template for the production of viral RNA, making the virions able to be produced in an endless stream and preventing their elimination by current drugs [7,8]. Given the limitations of current anti-HBV therapeutic drugs and the complex, long-term natural history of chronic HBV infection, developing precise diagnosis markers is crucial. These markers can effectively guide the selection of treatment strategies, predict disease prognosis in advance, and facilitate the clinical management of CHB. In the past decade, several studies have uncovered new markers specific to HBV, including HBV RNA and hepatitis B core-related antigen (HBcrAg) [9]. The HBcrAg is a composite marker of viral pre-core/core ORFs encoding proteins, including HBcAg, HBeAg, and p22cr. Moreover, the new roles of previously established markers at quantitative levels, such as quantitative hepatitis B surface antigen (qHBsAg), E antigen (qHBeAg), and quantitative hepatitis B core antibody (qAnti-HBc), have also been revealed.

**Figure 1:**
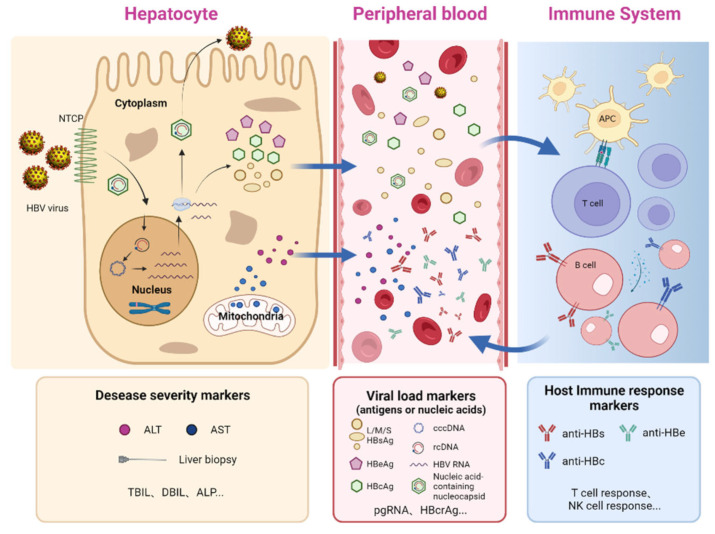
Biomarkers associated with the HBV life cycle and immune response. The figure illustrates the formation process and interrelationship of various markers associated with chronic HBV infection. The upper part of the figure is divided into three regions that represent events and factors in the liver, peripheral blood, and immune system, while the lower half classifies the biomarkers at different dimensions. ALT, alanine transaminase; AST, aspartate aminotransferase; TBIL, total bilirubin; DBIL, direct bilirubin; ALP, alkaline phosphatase; L/M/S HBsAg, large/middle/small surface antigens; NTCP, the cellular receptor for HBV; APC, antigen-presenting cell (created with BioRender.com).

In the clinic, both classic and emerging biomarkers are used to characterize HBV infection status at various dimensions in associating with the HBV life cycle and natural history of HBV infections (Figure 1). Specifically, the serum activities of hepatic enzymes, such as ALT/AST, are surrogates for hepatic inflammation but are not specific to HBV. Virological markers, including intrahepatic cccDNA, serum HBsAg, HBcrAg, HBV DNA, and HBV RNA, primarily reflect viral “loads” at various aspects. Accurately measuring cccDNA can be achieved through droplet digital PCR (ddPCR) assays, which use hepatocyte samples obtained through liver biopsy. However, the high cost of ddPCR and the invasive nature of liver biopsy limit the widespread use of cccDNA detection in routine clinical practice. Recently, serum HBV RNA has been proposed as a promising marker for assessing intrahepatic cccDNA activity or load and is useful in guiding the safe discontinuation of NAs therapy [10,11]. Unfortunately, the lack of standardized methods for measuring serum HBV RNA currently limits its clinical utility [12,13]. Moreover, the HBcrAg was also suggested to be an additional serum surrogate for intrahepatic cccDNA and positively associated with HBV rebound risk after treatment discontinuation [14,15]. Among serological antibody markers, the qAnti-HBc should be considered a universal surrogate marker for host antiviral immunity in chronic HBV infections (Figure 1). In 2013, our group first uncovered the qAnti-HBc level positively correlated with ALT activities and treatment response using a double-sandwich total anti-HBc immunoassay [16]. Subsequently, several cohort studies further confirmed the significance of quantitative anti-HBc measurements as a non-invasive marker for HBV-related liver inflammation and a predictor of treatment response in CHB patients who received interferons or NAs. The present review summarizes the current insights into the dynamic changes, clinical significance, and immune-related mechanisms of qAnti-HBc in different phases of HBV infections to explore the clinical utility of this new marker as a surrogate for host antiviral immune activity.

## 2. The qAnti-HBc Levels in the Natural History of HBV Infection

Both T cells and serum antibodies could theoretically be potential biomarkers of host anti-HBV immunity. However, virus-specific T cells are typically exhausted in cases of chronic HBV infections and pose challenges in detection [17,18]. Furthermore, detecting T cells requires intricate and complex cell culture procedures that are difficult to execute in clinical practice. For serological antibody markers, the antibodies against the surface antigen (anti-HBs), the E antigen (anti-HBe), and the core antigen (anti-HBc) are classical humoral immunity markers that are widely clinically used. However, the anti-HBs only appear after HBsAg loss, which indicates HBV infection is completely resolved, whereas the anti-HBe is just present in partial patients, often in those who have achieved HBeAg loss, and is usually associated with HBV DNA suppression. In contrast, the anti-HBc is detectable in nearly all phases of HBV infections and thereby potentially serves as a universal anti-HBV immune surrogate. The HBV core antigen (HBcAg) is an essential component for viral capsid, formatting an icosahedral (T = 3 or 4) particulate structure. The HBcAg is an essential component for viral capsid, formatting an icosahedral (T = 3 or 4) particulate structure. In contrast to the HBeAg, which shares approximately 152–154 identical amino acids but forms a secretable dimer protein, the HBcAg is remarkably more immunogenic in stimulating cellular and humoral immunity [19,20]. In addition, several studies suggested that HBcAg could stimulate TLR signaling to produce proinflammatory cytokines, whereas HBeAg plays the opposite role [20,21,22,23]. Traditionally, anti-HBc is regarded as the indicator for past or ongoing HBV infections and is used as a blood screening test in HBV low-to-medium epidemic regions [24,25]. Notably, the anti-HBc level in most individuals with persistent, even resolved HBV infection usually exceeds the upper limit of routinely used qualitative anti-HBc assays. Studies on the value of anti-HBc levels in chronic HBV infection require serial dilutions or assays with wide dynamic ranges to know the accurate difference of anti-HBc among different patients or the changes during the disease course, which cannot be determined by using qualitative assays.

### 2.1. High-Level of qAnti-HBc in Immune-Active Phases

In chronic HBV infections, individuals in the immune-active phase (also called the CHB phase) with active hepatitis typically exhibit ~10-fold higher levels of qAnti-HBc compared to those in the immune-tolerant or inactive-carrier phases when hepatitis is absent [26]. Several studies noted the positive correlation between serum ALT activity and qAnti-HBc in CHB patients, suggesting the latter may indicate hepatic inflammation [26,27,28]. Moreover, qAnti-HBc has been documented to correlate positively with the histological severity of hepatic inflammation determined by liver biopsy [28,29,30]. Interestingly, some studies have found that qAnti-HBc is also associated with fibrosis severity and can provide improved value for the performance of non-invasive indexes in combination with other parameters [31,32,33]. However, the correlation between qAnti-HBc and fibrosis degree is usually weaker than that with inflammation, suggesting that the former linkage may be partially attributed to the association of qAnti-HBc and liver inflammation. Since hepatic inflammation generally results from activated immune-mediated liver injury in chronic HBV infection, the qAnti-HBc reflects the host immune response against the virus. Consistent with the high level of serum qAnti-HBc, peripheral core-specific memory B cells were also found with significantly higher frequencies in immune-active CHB phases than those in immune-tolerant or inactive carriers [34]. HBcAg can induce both T-dependent and T-independent humoral responses [35]; though some human naïve B cells can be directly activated by HBcAg [36], HBcAg-specific helper T (Th) cells can promote anti-HBc producing B-cells [37,38]. On the other hand, HBcAg-specific B cells can act as antigen-presenting cells to facilitate T-cell response [39]. To summarize, a high level of qAnti-HBc in immune-active CHB phases is associated with hepatic inflammation and ALT elevation, reflecting the host’s anti-HBV immune activity.

### 2.2. The qAnti-HBc in Chronic HBV-Infected Individuals with Normal ALT

Liver tissue biopsy is the gold standard for diagnosing liver inflammation but is not commonly used due to its invasive nature [32]. The commonly used marker for liver damage, ALT, carries a risk of missed detection, with 20–30% of patients with normal ALT experiencing moderate to severe liver inflammation. Extensive studies have investigated the additional role of qAnti-HBc in discriminating inflammation and immune activation status in individuals infected with HBV and with normal ALT levels [29,40,41,42]. In a multicenter study, Zhang et al. revealed a dose-responsive relationship between qAnti-HBc and liver inflammation severity in 1376 untreated CHB patients with normal ALT (β = 0.48, *p* < 0.001), with the qAnti-HBc cut-off value for diagnosing moderate and severe inflammation at about 4.5 log10 IU/mL [41]. This study also noted that the decline of qAnti-HBc after NAs-treatment correlated with the alleviation of liver histological inflammation. In addition, Feng et al. also reported that elevated qAnti-HBc in CHB patients with normal ALT was associated with significant liver injury, although the cut-off value appeared to be lower in this study (3.7 log10 IU/mL) than that in the former-mentioned study [43]. Specifically, among HBeAg-negative chronic HBV-infected populations with normal ALT, a high level of anti-HBc was also found to be associated with significant liver inflammation [44].

Furthermore, a longitudinal study with long-term follow-up (median 19.8 years) in 182 HBeAg-positive children with normal ALT demonstrated that the baseline qAnti-HBc (500 IU/mL) is a strong independent predictor for spontaneous HBeAg seroconversion [45]. This study clearly characterized qAnti-HBc dynamics in the early CHB stage in children, showing an increase in qAnti-HBc before ALT rising and HBeAg seroclearance. On the other hand, the lower qAnti-HBc observed in this study than in adults with CHB may suggest a less intense anti-HBV immune response in children. Based on currently available evidence, CHB patients in the immune-tolerant phase with elevated qAnti-HBc levels, despite having no elevation in ALT, are highly likely to be associated with the presence of activated immune and hepatitis activity.

### 2.3. The qAnti-HBc in Occult or Resolved HBV Infections

In cases where HBsAg clearance is achieved, anti-HBc is often the only detectable marker of a past HBV infection. However, in HBsAg-negative individuals during the late phase of either occult or resolved HBV infections, the qAnti-HBc is typically around 1000-fold lower than that observed in HBsAg-positive cases [46]. In transplant donors with occult HBV infection, which is defined as the presence of intrahepatic HBV DNA but the absence of serum HBsAg, a relatively higher anti-HBc level (>4.4 COI) was associated with the cccDNA presence evidenced by ddPCR [47]. A cross-sectional study also found a qAnti-HBc level over 6.6 IU/mL is associated with occult HBV among HBsAg-negative individuals [46]. Moreover, Yang et al. discovered that in patients with lymphoma and resolved HBV infection who received rituximab-containing chemotherapy, a qAnti-HBc level above 6.41 IU/mL was independently linked with an increased risk of HBV reactivation [48]. In addition, among patients who experienced HBV reactivations, the qAnti-HBc markedly increased following HBV DNA rebound and hepatitis flare [48]. A recent preprinted study also reported similar findings [49]. These findings suggested that the high qAnti-HBc presence in the HBsAg-negative phase may indicate the presence of non-eradicated HBV residuals.

### 2.4. The qAnti-HBc in Patients with HBV and Other Hepatotropic Pathogens Co-Infections

The established relationship between elevated qAnti-HBc and activated hepatitis in HBsAg-positive individuals raises an interesting question about the qAnti-HBc level in individuals co-infected with HBV and other hepatotropic pathogens. However, current understanding on this topic is limited. Some studies have characterized qAnti-HBc in HBV/HDV superinfections. In a study of a small number of patients with ALT elevation, qAnti-HBc was found at lower levels in HDV-superinfected inactive carriers than in untreated CHB patients [50]. Multivariate analysis in 122 HDV genotype 1 and HBV genotype D co-infected patients suggested that there was no independent association between serum qAnti-HBc and HDV-related disease for either ALT elevation or cirrhosis [51]. The median qAnti-HBc level in HDV/HBV superinfections was less than 1500 IU/mL, which is close to that among inactive HBV carriers observed in other studies. These findings suggest elevated qAnti-HBc is specifically associated with hepatitis caused by HBV-relative immune activity [52]. Therefore, qAnti-HBc may potentially differentiate disease causes in chronic HBV carriers co-infected with other hepatotropic pathogens (such as HCV or HEV) or co-existing non-infectious liver diseases, and further investigations are warranted.

## 3. Prognostic and Predictive Roles of qAnti-HBc in Chronic HBV Infection

### 3.1. The qAnti-HBc Predicts HBeAg Seroconversion

Extensive studies demonstrated that the high level of qAnti-HBc is a strong predictor for HBeAg seroconversion. For HBeAg-positive individuals chronically infected with HBV, HBeAg seroconversion (ESR) often confers a favorable prognosis with reduced incidence of progressive liver inflammation and advanced liver diseases [53,54,55]. Our early study uncovered that higher qAnti-HBc in HBeAg-positive CHB patients had more probability of achieving ESR after treatments with adefovir dipivoxil (ADV) or PEG-IFN [4]. Notably, the optimal cut-off value was determined as 29,000 IU/mL (4.46 log10) for the ADV-treated group and 9000 IU/mL for the PEN-IFN group in this study. Using a retrospective multicenter cohort study consisting of 231 and 560 patients receiving PEG-IFN and NAs (Telbivudine, alone or plus TDF), Fan et al. confirmed the baseline qAnti-HBc could independently predict the ESR at the endpoint of treatment with a cut-off value of 4.4 log10 IU/mL [56]. Another study by Hou et al. also found pretreatment qAnti-HBc (>30,000 IU/mL) was a strong predictor for ESR in the PEG-IFN regimen [57]. The same study also found that higher qAnti-HBc indicated a higher probability of achieving a virological or combined response for PEG-IFN-based therapy [57]. Among patients with long-term entecavir regimens, the qAnti-HBc (>4.65 log10 IU/mL) was also a strong predictor for ESR at either weeks 144 (~3 years) or 240 (~5 years) after treatment administration [58]. In addition to the above-mentioned studies, several recently published studies performed in HBeAg-positive patients with various antiviral treatments also reported consistent results [59,60,61,62,63].

In the natural course of chronic HBV infection, similar to that observed in the anti-HBV treatment cohorts, the qAnti-HBc also plays a significant predictive role for ESR. Liu et al. showed the independent association between high levels of qAnti-HBc with spontaneous ESR and suggested it is a surrogate marker of the anti-HBV T-cell activity in retrospective analyses based on the community-based REVEAL-HBV cohort [64]. In addition, as mentioned above, in the HBeAg-positive children’s cohort study, Chen et al. demonstrated that a high level of qAnti-HBc was predictive for spontaneous ESR [45]. Moreover, before ESR was achieved, children’s serum anti-HBc levels gradually increased with age and ongoing liver inflammation [45]. In summary, a higher qAnti-HBc is a biomarker with more ESR probability associated with a favorable outcome in the HBeAg-positive CHB stages.

### 3.2. Relationship between qAnti-HBc and Viral Clearance in HBeAg-Negative Individuals

Unlike that in HBeAg-positive people, the prognostic significance of qAnti-HBc in HBeAg-negative CHB phases was not fully understood. However, some studies suggested the prognostic direction for this marker in HBeAg-negative populations was different from that in HBeAg-positive patients. In a retrospective analysis based on the REVEAL-HBV cohort (*n* = 2500), a lower level of qAnti-HBc (<3.0 log10 IU/mL) in HBeAg-negative patients was independently associated with a higher HBsAg seroclearance (53% vs. 19.8%) after a 12-year follow-up. A combination of the qAnti-HBc and HBsAg levels presented improved performance of serological parameters in predicting HBsAg seroclearance in HBeAg-negative CHB patients [65]. In addition, some other studies performed in patients who received antiviral treatments with PEG-IFN and/or NAs also suggested the qAnti-HBc negatively correlated with HBsAg seroclearance [66,67]. Since data from individuals with occult or resolved HBV infections are largely lower qAnti-HBc levels compared to HBsAg-positive populations, it is reasonable to expect a low qAnti-HBc level in HBeAg seroconverts reflects successful immune control against the virus and thereby associates with an increased chance of HBsAg loss and favorable outcome. Notably, among CHB patients with NAs regimens, some studies found that a lower qAnti-HBc at the end of treatment is associated with a higher risk of off-treatment clinical relapse [68,69]. Although these studies were conducted on small populations of both HBeAg-positive and -negative individuals, their findings appear to conflict with the relationship between a lower qAnti-HBc and a higher chance of HBsAg seroclearance. Differences in sample composition and length of follow-up may account for the potential discrepancy between these studies. However, another possible explanation is that patients who experience clinical relapse (virological rebound with ALT elevation) after drug discontinuation often exhibit ALT flare, which is linked to immune-related injury of HBV-infected hepatocytes and a subsequent high probability of HBsAg loss [70,71]. The systematical investigation of off-treatment qAnti-HBc changes in NA-treated CHB patients, particularly for HBeAg-negative populations, would be helpful. Nevertheless, further studies are needed to elucidate the predictive value of qAnti-HBc in HBeAg-negative CHB fully.

### 3.3. Prognostic Potentials of qAnti-HBc in HBV-Related ACLF and OLT Patients

Li et al. investigated the prognostic potentials of qAnti-HBc in patients with HBV-related acute-on-chronic liver failure (ACLF). This study found the HBV-ACLF patients had a significantly higher level of anti-HBc than CHB patients (4.95 ± 0.54 vs. 4.47 ± 0.84 log10 IU/mL), and the qAnti-HBc showed a comparable and complementary prognostic accuracy to MELD (model for end-stage liver disease) score in predicting clinical outcome (survival or death). A high level of qAnti-HBc was associated with less mortality in HBV-ACLF patients [72]. In another study conducted in HBV-infected patients who received orthotopic liver transplantation (OLT), Lou et al. also suggested a high level of pre-transplantation qAnti-HBc was associated with a low risk of post-transplantation HBV recurrence [73]. These studies suggested prognostic potentials of qAnti-HBc for clinical outcomes in HBV-related ACLF and OLT patients, which merits further validation.

## 4. Virological and Immunological Regulation of qAnti-HBc

**Figure 2:**
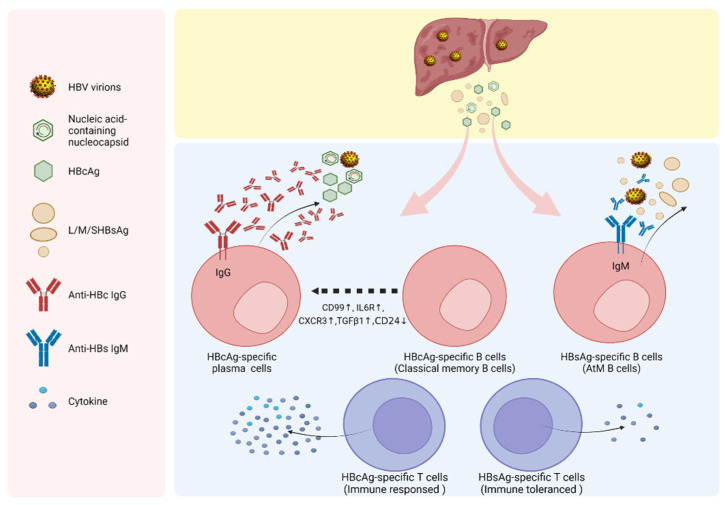
Characteristics of the immune response against hepatitis B core and surface proteins. The HBcAg-direct immune response appears to be more activated than that against HBsAg at both the T-cell and B-cell levels. The high levels of HBsAg may cause T-cell exhaustion and B-cell dysfunction. HBsAg-specific B cells show an atypical memory phenotype (AtM) in CHB. In contrast, HBcAg-specific B cells are in a more normal state, with more active antibody production and secretion. The HBcAg stimulates anti-HBc antibody vial T-dependent and T-independent pathways, and the qAnti-HBc level correlates with the HBcAg-direct B cell (created with BioRender.com).

The well-established relationship between qAnti-HBc and HBeAg seroconversion implies the linkage between activated core-specific immunity in mediating HBeAg loss and virological control. Peripheral high levels of HBsAg and HBeAg are essential viral factors to induce T cell exhaustion and immune tolerance in persistent HBV infections. For HBeAg, the animal study suggested that maternal-derived HBeAg alters hepatic macrophages toward M2 polarization and impaired CD8+ cytotoxic T lymphocyte (CTL) response leading to viral persistence [74]. In CHB patients, the HBeAg can also cause the expansion of myeloid-derived suppressor cells (MDSCs) to suppress the proliferation and function of T cells [75]. In addition, the presence of TIR BB-like in HBeAg aa(-10)-(-1) region (absent in HBcAg) may interact and disrupt TLR-mediated signaling, thereby evading innate immune response [76]. Overall, extensive evidence supports the immune suppression role of HBeAg in HBV persistence. As highly homologous proteins, the HBcAg and HBeAg share approximately 149–154 amino acids and inevitably have several cross-reactive T-cell and B-cell epitopes. Elevated serum levels of anti-HBc may imply the activations of HBcAg-directed and HBeAg cross-reactive B cell responses. The increased anti-HBc-producing B cells can also facilitate antigen presentations to promote helper and cytotoxic T cell response, mediating immune clearance of hepatocytes with HBcAg and/or HBeAg expressions [77]. On the other hand, high levels of anti-HBc antibodies may also play a direct cytotoxic effect via Fc-mediated immune modulations, such as complement-dependent cytotoxicity (CDC) [78,79,80]. For either pathway, patients may experience subsequent liver injury and hepatic inflammation, which account for the relationship between qAnti-HBc and hepatitis activity. Previous studies have found that HBc-specific T cells are more activated, while HBs-specific T cells are significantly depleted in HBV-specific T cells of CHB patients by peptide-based culture-based ELISPOT, which may be caused by long-term high levels of HBsAg and HBV DNA [77]. In recent years, several studies investigated the roles of antigen-specific B cells in chronic HBV infections. Although the frequencies of HBsAg-specific B cells in CHB patients appeared to be comparable with that observed in HBV-vaccinated healthy controls, these B cells were found to be enriched in an atypical memory B cell (AtM) phenotype, which was dysfunctional for survival, expansion, and anti-HBs production [81]. By contrast, consistent with serological antibody levels, peripheral HBcAg-specific B cells were more activated, with higher frequency than HBsAg-specific B cells, and less AtM phenotype [82]. Moreover, a direct link between high levels of qAnti-HBc and more HBcAg-specific B-cells among patients in immune-activated phases has been evidenced [34]. The characteristics of the immune response against HBcAg and HBsAg in chronic HBV infections are illustrated in Figure 2.

The marked contrast in qAnti-HBc between HBsAg-positive populations and individuals with resolved HBV infection [46], as well as the gradual decline in qAnti-HBc and HBcAg-specific B cells in CHB patients observed during antiviral therapy [13,35], implies that viral antigen is crucial for the stimulation and maintenance of anti-HBc. Nonetheless, the low levels of qAnti-HBc observed in immune-tolerant patients with high HBV replication and antigen load indicate that host immune status also plays an essential role in regulating qAnti-HBc in chronic HBV infection [46]. However, how and when the anti-HBc level initially increases in the immune-tolerant stage is still largely unknown. In addition, the causal relationship between qAnti-HBc elevation and liver inflammation occurrence in CHB remains to be explored. The HBcAg may induce the rapid proliferation of certain naïve B cells to produce some anti-HBc via a T-independent pathway during the early immune encounter. Based on classical understandings, as a virion component, HBcAg is contained within envelopes and is not readily accessible by B cells in a normal situation. Therefore, it seems reasonable to assume liver damage occurs before qAnti-HBc elevation, as damaged hepatocytes can release non-enveloped HBcAg to stimulate antibody production. Nevertheless, a long-term (median follow-up for 19.8 years) longitudinal study suggested the qAnti-HBc increase precedes ALT rising among HBeAg-positive children [45], implying there is an alternative driving for initial qAnti-HBc elevation. Notably, some studies revealed that naked HBcAg capsid could also be secreted into the cell culture supernatants and is evidenced as non-enveloped capsid-antibody complexes (CACs) in CHB patients’ sera [83,84]. Based on the above-mentioned evidence, we conjectured that the secreted naked HBcAg capsid is potentially responsible for triggering anti-HBc rising and subsequent starting conversion from the immune tolerance into the immune clearance phase during CHB natural course (Figure 2). To some extent, this hypothesis agreed with the theory proposed by Zhang et al. that the capsid-derived particles release is an indispensable trigger for HBV-related immunopathology [85]. Although current insights for regulating naked HBcAg capsid release are still less understood, the large surface protein (the level or the ratio of LHBs/sHBs) and HBcAg mutations could affect the naked capsid secretion in HBV cell cultures [86,87]. Further studies on the mechanisms and triggers that lead to changes in qAnti-HBc during the early immune-tolerant phase of HBV infection and its potential relationship with naked HBcAg secretion could offer valuable insights into HBV pathogenesis and also inform the developments of innovative HBV therapies.

## 5. Conclusions and Future Perspectives

**Figure 3:**
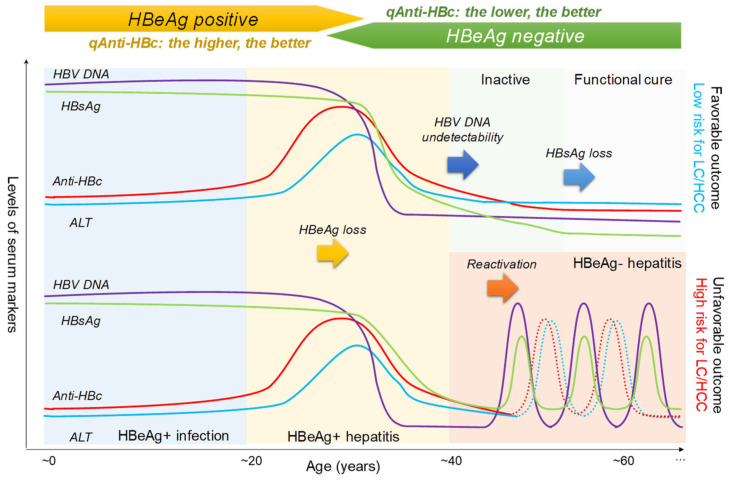
Schematic representation of the dynamic changes in the levels of the typical serological markers during chronic hepatitis B virus infection. The CHB natural history is divided into periods according to the infection outcome and disease progression, which are indicated by different color blocks. The dynamic changes of HBV DNA (purple), HBsAg (green), qAnti-HBc (red), and ALT (blue) in the CHB course infection are shown. The qAnti-HBc has different implications according to the HBeAg status and increased HBV persistence time.

Extensive studies uncovered the new roles of qAnti-HBc in the CHB natural course and treatment process. Although further excavations and validations are still required to be conducted, based on currently available evidence, the clinical significance of qAnti-HBc could be summarized as following points for reference (also schematically presented in Figure 3):

(1) The qAnti-HBc is a well-validated predictor for spontaneous and treatment-induced HBeAg seroconversion among HBeAg-positive individuals. People with a higher level of qAnti-HBc have more chance of achieving HBeAg seroconversion. The cut-off values from different studies showed a little difference, but 4.0–4.5 log10 (about 10,000–30,000) IU/mL is possibly preferable for adult CHB patients.

(2) For individuals in HBeAg seronegative phase with successful virological control, a persistent low level (maybe <1000 IU/mL) of qAnti-HBc links with a high probability of achieving HBsAg loss.

(3) Regardless of HBeAg status or ALT activity, the qAnti-HBc (>4.0 or >4.5 log10 IU/mL) positively correlates with the activated host anti-HBV immune activity and the risk of the presence of significant hepatic inflammation.

(4) During the recovery phase of post-HBsAg loss, qAnti-HBc may reflect the non-eradicated HBV residuals, and a relatively higher qAnti-HBc indicates the risks for occult HBV infection or HBV reactivation when receiving rituximab-containing chemotherapy.

In conclusion, the qAnti-HBc is a surrogate marker for host anti-HBV immune activity in chronic HBV infections. Elevated qAnti-HBc-associated antiviral immunity is essential to drive immunological control against HBV infection during HBeAg-positive phases but signifies immune-escape hepatitis during HBeAg-negative phases. Wide clinical applications of qAnti-HBc are still challenged by the absence of standardized automatic assays and the well-validated cut-off index for various scenarios [88]. Further exploration of the clinical implications of qAnti-HBc will facilitate the optimization of CHB management and enhance our understanding of the immune mechanisms and virus–host interactions in chronic HBV infections. Moreover, more extensive studies are warranted to elucidate the immunological and virological mechanisms that govern qAnti-HBc dynamics.

## Data Availability

Not applicable.
